# Know Thy Model: Charting Molecular Homology in Stromal Reprogramming Between Canine and Human Mammary Tumors

**DOI:** 10.3389/fcell.2019.00348

**Published:** 2019-12-17

**Authors:** Enni Markkanen

**Affiliations:** Institute of Veterinary Pharmacology and Toxicology, Vetsuisse Faculty, University of Zurich, Zurich, Switzerland

**Keywords:** laser-capture microdissection, RNA sequencing, canine mammary carcinoma, canine mammary adenoma, breast cancer, tumor stroma, tumor microenvironment, comparative oncology

## Abstract

Spontaneous canine simple mammary tumors (CMTs) are often viewed as models of human breast cancer. Cancer-associated stroma (CAS) is central for initiation and progression of human cancer, and is likely to play a key role in canine tumors as well. Until recently, however, canine CAS in general, and in CMT in particular, lacked detailed characterization and it remained unclear how canine and human CAS compare. This void in knowledge regarding canine CAS and the resulting lack of unbiased cross-species analysis of molecular homologies and differences undermined the validity of the canine model for human disease. To assess stromal reprogramming in canine breast tumors, we have recently established a protocol to specifically isolate and analyze CAS and matched normal stroma from archival, formalin-fixed paraffin embedded (FFPE) clinical tumor samples using laser-capture microdissection followed by next-generation RNA-sequencing. Using this approach, we have analyzed stromal reprogramming in both malignant canine mammary carcinomas (mCAs) as well as benign canine mammary adenomas in a series of studies. Our results demonstrate strong stromal reprogramming in CMTs and identify high-grade molecular homology between human and canine CAS. Here, I aim to give a short background on the value of comparative oncology in general, and spontaneous CMT in particular. This will be followed by a concise review of the current knowledge of stromal reprogramming in both malignant canine mCA and benign adenoma. Finally, I will conclude with insights on highly conserved aspects of stromal reprogramming between CMT and human breast cancer that accentuate the relevance of CAS in CMT as a model for the human disease.

## Introduction

The majority of all cancers derive from corrupted epithelial cells that give rise to tumor cells that disregard the tissue boundaries of their natural habitat. Yet, these epithelial tumor cells are not living in an isolated environment, and – far from being self-sufficient – heavily depend on their microenvironment for survival and growth (Hanahan and Coussens, 2012). This microenvironment, also called cancer-associated stroma (CAS), consists of a heterogeneous mixture of different non-tumor cells (among them fibroblasts, immune cells, vascular cells, adipocytes, and others), as well as extracellular matrix (ECM). CAS has been abundantly shown to play a key role in initiation and progression of a wide variety of tumors, and its manifold roles in tumor biology have been widely documented [e.g., reviewed in Bissell and Hines (2011); Bussard et al. (2016), Hanahan and Coussens (2012); Kalluri (2016), and Quail and Joyce (2013)]. Nevertheless, the field is still far from completely understanding the mechanisms by which CAS influences tumor biology, the molecular players that are involved, and the intricacies of the cross-talk between CAS and tumor cells.

Due to the closely related pathophysiology, naturally occurring cancers in the domestic dog are progressively leveraged as a valuable source of information to better understand the biology behind tumor development and possibly find novel anti-cancer treatments (Karlsson and Lindblad-Toh, 2008; Gardner et al., 2015; Rogers, 2015). While increasing efforts have been focused on analysis of the molecular aspects of tumor cells in canine cancers and their comparison with aberrations in human tumor cells, canine CAS greatly lacks characterization. Hence, it remains completely unclear how canine and human CAS compare. Given the central importance of CAS for the biology of human cancer, this striking shortage of data on canine CAS and the resulting lack of unbiased cross-species analysis of molecular homologies and differences threaten to undermine the validity of the canine model for human disease.

Among various other tumor types, naturally occurring canine mammary tumors (CMTs) are viewed as excellent translational model for human breast cancer. Recent work by my group has begun to assess stromal reprogramming in spontaneous CMTs by next-generation RNA-sequencing (RNAseq) in both malignant canine mammary carcinomas (mCAs) and benign canine mammary adenomas, and to probe the extent of molecular homology between human and canine CAS. In the following, I aim to give a short background on the value of comparative oncology in general, and spontaneous CMT in particular. This will be followed by a concise review of the current knowledge of stromal reprogramming in both malignant canine mCA as well as benign adenoma. Finally, I will conclude with insights on highly conserved aspects of stromal reprogramming between CMT and human breast cancer that accentuate the relevance of CAS in CMT as a model for the human disease.

## Naturally Occurring Tumors in Dogs as Translational Models for Human Cancer

A plethora of *in vitro* and *in vivo* models have been used over the last century to gain insights into cancer biology. While these models have undoubtedly been highly informative in many aspects and lead to various scientific breakthroughs, the inherent limit in most of the used models is their inability to fully replicate the conditions and mirror the complexity of spontaneously developing patient tumors (Karlsson and Lindblad-Toh, 2008; Uva et al., 2009; Rowell et al., 2011; Liu et al., 2014; Schiffman and Breen, 2015). The field of comparative oncology aims to address these shortcomings by widening the research focus from classical rodent models toward spontaneous tumors that develop in other animals, such as the domestic dog. This additional perspective is perceived as a chance to complement and enhance our understanding of complex diseases, such as cancer, as the comparison of tumor development and risk factors across species provides the opportunity to discover basic mechanisms of tumorigenesis (Karlsson and Lindblad-Toh, 2008; Paoloni and Khanna, 2008; Gordon et al., 2009; Schiffman and Breen, 2015). Due to the many similarities shared between dogs and humans, the domestic dog is considered one of the best examples for comparative oncology. Firstly, the number of genes in dogs and humans are comparable, and evolutionarily conserved alterations in the genome are shared between these species (Bejerano et al., 2004; Karlsson and Lindblad-Toh, 2008; Rivera and Euler von, 2011; Rowell et al., 2011; Schiffman and Breen, 2015). Cancer in both species develops spontaneously with similar pathophysiology, and often manifests in similar clinical presentation and histology. As such, development of spontaneous tumors in dogs has strong parallels with the natural progression of cancer development in humans, and is considered a better proxy of tumor biology than animal models with induced tumorigenesis. The higher life expectancy compared to rodent models and the same environmental factors that dogs and humans are exposed to, combined with the fact that dogs often receive a high level of healthcare, further strengthen the value of comparatively analyzing canine and human cancers. Also, as a result of inbreeding and high degrees of consanguinity, certain breeds of dogs have been shown to carry genetic predispositions for certain cancer types, facilitating the discovery of risk alleles responsible for the disease [reviewed in Schiffman and Breen (2015)]. Altogether, these insights emphasize the potential of the dog as models for human cancer and offer the possibility to overcome limits of xenograft and genetically engineered rodent models leading to improved understanding of tumor biology and biomarker discovery. The interested reader is further referred for a more detailed discussion to several excellent reviews on the subject (Rowell et al., 2011; Schiffman and Breen, 2015).

## Canine Mammary Tumors as a Model for Human Breast Cancer

Among many different cancer types, especially CMTs have garnered attention as useful models for human breast cancer (Sorenmo et al., 2009; Queiroga et al., 2011b; Abadie et al., 2017; Nguyen et al., 2017). In relative terms with regards to life expectancy (i.e., converting “dog years” into “human years”), the age of onset is comparable between women and bitches. The incidence of CMTs starts to increase after the age of 6 years (the equivalent of age 40 years in humans) and peaks between 8 and 14 years (humans age 50–70 years) (Queiroga et al., 2011b; Rowell et al., 2011; Bundesamt für Statistik, 2015; Bray et al., 2018; World Health Organization, 2018). Furthermore, it is the most frequent cancer diagnosed both among female dogs as well as women suffering from cancer (Sorenmo et al., 2009; Lahkhani et al., 2012; Liu et al., 2014; Bray et al., 2018). A retrospective study on canine tumors in Switzerland between 1955 and 2008 found that 20.5% of all canine tumors were located in the mammary gland (Grüntzig et al., 2015). A retrospective study in Italy between 1985 and 2002 even found 70% of all tumors of female dogs to be located to the mammary gland (Merlo et al., 2008). On a global level, CMTs occur in >40% of female dogs and show and annual incidence rate varying between 192 and 205/100,000 dogs, comparable to human data with incidence rates of 125/100,000 women in the United States, and 144/100,000 women in Switzerland (Queiroga et al., 2011b; Sleeckx et al., 2011; Liu et al., 2014; Bundesamt für Statistik, 2015). Interestingly, CMT incidence is lower in the United States than in other countries like Sweden, presumably because dogs tend to get neutered at an early age in the United States and therefore receive less gestagen preparations for heat prevention (Sleeckx et al., 2011).

The anatomy of the normal mammary gland is similar in dogs and women. The alveoli and ducts of the mammary gland consist of luminal epithelial cells lined by myoepithelial cells and are separated from the surrounding connective tissue by the basement membrane (Liu et al., 2014; Santos and Matos, 2015). In both species tumor formation is seen as a dynamic process starting from benign hyperplastic lesions that can evolve into a carcinoma *in situ.* In a further step, these tumors can become invasive, which is marked by the disruption of the basement membrane and potential seeding of metastases (Gilbertson et al., 1983; Burstein et al., 2004; Simpson et al., 2005; Sorenmo et al., 2009). On a molecular level, many of the key alterations in human breast cancer are faithfully recapitulated in CMTs, including germline mutations in BRCA1, BRCA2, and TP53 that are associated with an enhanced risk of hereditary cancer in humans (Liu et al., 2014; Matos and Santos, 2015; Santos and Matos, 2015; Schiffman and Breen, 2015). And finally, besides clinical factors such as tumor size, lymph node involvement, and clinical stage, the prognostic value of histo-pathological aspects such as tumor type and grade, and molecular subtypes (luminal A, luminal B, HER2-enriched, and basal-like) is conserved between canine and human breast cancer (Rivera et al., 2009; Queiroga et al., 2011b; Sleeckx et al., 2011; Lahkhani et al., 2012; Rasotto et al., 2012, 2017; Im et al., 2013; Pena et al., 2013; Nguyen et al., 2017). However, assessment of molecular subtypes is still limited to research purposes and not routinely applied in CMTs (Sleeckx et al., 2011; Rasotto et al., 2017).

Altogether, the similarities between CMTs and human breast cancer suggest wide-ranging homologies in tumor biology. Canine studies offer the opportunity to find novel biomarkers not only for veterinary use, but also to benefit human patients. Comparing the same disease in two different species additionally helps differentiating the molecular “drivers” of the disease from mere “passengers,” as key pathways should be conserved between species. Finally, clinical trials in dogs can be conducted in a shorter period than human studies, due to a reduced lifespan and associated earlier manifestation of cancer (Karlsson and Lindblad-Toh, 2008).

## Largely Uncharted Territory: Stromal Reprogramming in Canine Mammary Tumors

While the importance of CAS in cancer initiation and progression is becoming increasingly clear, data regarding the molecular composition of CAS in canine cancer overall, and CMTs in particular are sparse. Traditionally, analysis of tumor samples by RT-qPCR or next-generation sequencing approaches is performed in bulk. While highly informative, the major drawback of bulk tissue analysis is the fact that results reflect the mixture of all cells present in the sample, not discriminating between epithelial cancer cells and other non-neoplastic cells. The avoidance of inclusion of samples “too rich in stroma” is usually attempted by setting a cut-off value for stromal content as exclusion criterium. Nevertheless, results from such bulk analyses clearly are a conglomerate of highly varying amounts of different cell populations present at sampling. Thus, this approach heavily complicates the correct attribution of observed changes either to the cancer cells or to the stromal cells. To date, most studies investigating CMT on a molecular level, such as by sequencing or microarray analysis, have analyzed tumor tissue in bulk (e.g., Uva et al., 2009; Klopfleisch et al., 2010, 2011; Liu et al., 2015; Bulkowska et al., 2017). Accordingly, specific analysis of CAS in CMTs has thus far been restricted to just single markers that were analyzed predominantly through immunohistochemistry (IHC). CAS is composed of various different cellular and extracellular components that have been shown to exhibit strong influence on the hallmarks of tumor cells (Hanahan and Coussens, 2012). Of these, in CMTs, most attention has been focused on the roles of cancer-associated fibroblasts (CAFs), a few components of the ECM, a subset of infiltrating immune cells, as well as single markers for angiogenesis. In the following, I will attempt to shortly summarize the currently available data on the state of these components in CAS of CMTs.

### Cancer-Associated Fibroblasts and the Extracellular Matrix

Cancer-associated fibroblasts are a heterogeneous population of activated fibroblastic cells that present the most abundant cell fraction in CAS and strongly influence tumor development and progression (Chen and Song, 2019). CAFs often stain positively for alpha smooth muscle actin (αSMA), a marker for myofibroblast activation, and αSMA expression has been associated with reactive tumor stroma in human breast cancer and other tumors. As such, upregulation of αSMA is often used as marker for CAS, and enhanced expression of αSMA in human breast cancer is associated with poor prognosis (Sappino et al., 1988; Elenbaas and Weinberg, 2001; Yazhou et al., 2004; Surowiak et al., 2006, 2007; Yamashita et al., 2012). In CMTs, αSMA-positive myofibroblasts have been detected in CAS of adenomas and carcinomas, but not in normal breast tissue, increasing in abundance with higher tumor grade and histopathological signs of invasion and metastasis, and significantly related to poor prognosis (Yoshimura et al., 2011).

The main function of fibroblasts is to maintain the integrity of the ECM as structural support for cells and organs. As activated fibroblasts, CAFs strongly influence CAS composition and architecture through production and remodeling of ECM. Changes in collagen density and fiber organization have been associated with tumor grade and overall survival in CMTs (Case et al., 2017). Expression of the ECM molecule Tenascin-C (Tn-C), most likely produced by myofibroblasts, increased from benign adenomas to malignant carcinomas, and with signs of invasion and metastasis (Faustino et al., 2002; Yoshimura et al., 2011, 2014). Versican is another component of the ECM whose expression has been found to increase with malignancy and invasiveness of the tumor cells (Damasceno et al., 2016). Interestingly, the stroma of malignant tumors has been described to increasingly express the known multidrug resistance-causing transporters P-glycoprotein (PGP) and breast cancer resistance protein (BCRP), suggesting a role for the tumor stroma in the development therapeutic resistance (Levi et al., 2016). The most thoroughly investigated ECM-remodeling enzymes in CMTs belong to the group of ECM-degrading proteases including matrix metalloproteinases (MMPs) and urokinase-type plasminogen activator (uPA) and their inhibitors. These play a key role in ECM homeostasis in human breast cancer (Chen and Song, 2019). In CMTs, MMP2, MMP9, MMP14, and uPA levels increase from healthy tissue to benign to malignant CMTs, and are associated with increasing histological grade, signs of invasion, and early death from CMT, and present good prognostic factors (Papparella et al., 1997, 2002; Yokota et al., 2001; Hirayama et al., 2002; Kawai et al., 2006; Vinothini et al., 2009; Aresu et al., 2011; Lamp et al., 2011; Santos et al., 2011b, 2012, 2013; Santos and Matos, 2015). Unfortunately it is not always clear where exactly these MMPs were expressed, as some of the analyses were performed on bulk tumor tissue. Nevertheless, it is evident that MMP activity is mainly focused on the ECM, and a subset of these studies detected their expression also in fibroblasts close to the invasive tumor cells. Interestingly, MMP13 expression decreased significantly between benign and malignant CMT (Aresu et al., 2011).

The picture is slightly less clear for the MMP inhibitors: most reports have found TIMPs 1-3 and RECK to be highly expressed in malignant carcinomas (Papparella et al., 1997; Yokota et al., 2001; Hirayama et al., 2002; Kawai et al., 2006; Santos et al., 2011a). One report found expression of TIMP-2 to decrease in tumor tissue compared to controls, and also to decrease from grade I to grade III tumors (Vinothini et al., 2009), while Aresu et al. (2011) did not find statistically significant differences in TIMP-2 (nor TIMP-1, TIMP-3, or RECK) levels between benign and malignant tumors.

In summary, understanding of fibroblast activation and ECM remodeling in CMTs has thus far mainly focused on αSMA-positive myofibroblasts, and expression of Tn-C, MMPs, and their inhibitors. While these analyses have yielded interesting data also regarding similarities to CAS in human breast cancer, the understanding of both fibroblast activation and ECM remodeling in CMT remains extremely limited to date.

### Infiltrating Immune Cells

Infiltration of immune cells into tumors has been a longstanding area of interest in tumor biology, and the cellular composition of the immune infiltrate is clearly linked to disease outcome in CMTs (Gilbertson et al., 1983; MacEwen, 1990; Kim et al., 2013). High levels of CD4+ and CD3+ T-cells have been associated with metastasizing tumors and shorter overall survival (Estrela-Lima et al., 2010; Saeki et al., 2012; Carvalho et al., 2015a, b, 2016b). More detailed assessment of the localization of immune infiltrates with respect to the tumor cells found that tumor-infiltrating CD3+ T-lymphocytes were significantly more frequent in benign than malignant tumors, and conversely, peripheral CD3+ cells were more frequent in malignant than benign tumors (Carvalho et al., 2011). Furthermore, high number of neutrophils were associated with aggressive CMTs, while in contrast high amounts of plasma cells, macrophages, and CD8+ T-cells, together with low numbers of CD4+ T-cells, were associated with less aggressive tumors (de Souza et al., 2018). Taken together, these results suggest a strong role for T-lymphocytes in progression of CMTs, and also highlight that it is important to assess not only abundance of immune cells, but also identify their subtypes and define their exact localization within the tumor, as infiltrating immune cells that shielded from reaching the tumor cells cannot achieve immune control, and might really do more damage by fueling tumor-promoting inflammation instead. This is in line with current concepts of immunologically hot vs. cold tumors in humans (Galon and Bruni, 2019). Accordingly, presence of Foxp3+ regulatory T-cells (T-regs) and myeloid-derived suppressor cells (MDSCs) positively correlated with adverse prognostic factors, such as high histological grade, lymphatic invasion, and metastasis (Król et al., 2011a; Kim et al., 2012; Carvalho et al., 2016a; Mucha et al., 2016; Sakai et al., 2018). These findings support the concept that immune suppression through T-regs and MDSCs might contribute significantly to CMT progression.

Several studies have shown a strong correlation between high levels of tumor-associated macrophages (TAMs) and indicators of malignancy, metastasis, as well as worse overall survival in CMT (Restucci et al., 2002; Król et al., 2011b; Raposo et al., 2012, 2013; Lim et al., 2015; Carvalho et al., 2016b; Reis dos et al., 2019). All of these studies detected TAMs based on IHC detection of MAC387, but unfortunately did not attempt further subtyping of the macrophages into (antitumoral) M1 or (pro-tumorigenic) M2 phenotype. More detailed data regarding M1/M2 polarization of TAMs in CMT have been recently emerging, demonstrating significantly higher numbers of M2-TAMs in malignant CMTs while benign tumors harbored M1-TAMs, suggesting a M1-to-M2 shift of TAMs in malignant CMTs (Monteiro et al., 2018; Seung et al., 2018). While these results are highly interesting, there remains some controversy regarding whether CD204 represents a useful IHC marker for M2-polarized macrophages in dogs that awaits clarification (Belluco, 2018).

In summary, striking parallels between canine and human CAS with respect to the effect of the type of immune cell that strongly determines the effect on tumor progression are beginning to emerge. The interested reader wishing to further extend on parallels of CMT with human breast cancer in terms of tumor-associated inflammation is referred to a recent review on the topic (Carvalho et al., 2016c). Despite this progress, the field is still far from a complete understanding of the effects of different immune cells on the clinical course and prognosis of the disease and more detailed insights are needed to further clarify many of the outstanding questions. Further detailed insights into immune components in CAS of CMTs, ideally also with regards to molecular subtypes, are highly anticipated.

### Angiogenesis

Sustained angiogenesis represents one of the core hallmarks of cancer (Hanahan and Weinberg, 2011). A series of studies has assessed the contribution of blood vessel supply to the biology of CMTs. Indeed, in analogy to human breast cancer, increased microvessel density (MVD) correlated with malignancy and metastasis (Graham and Myers, 1999; Restucci et al., 2000; Millanta et al., 2006; Lavalle et al., 2009; Al-Dissi et al., 2010; Queiroga et al., 2011a; Carvalho et al., 2013; Sleeckx et al., 2014; Diessler et al., 2016; Anjos Dos et al., 2019).

A plethora of different molecules are involved in controlling the rate and extent of angiogenesis. Among the best studied ones are vascular endothelial growth factors (VEGFs) that regulate formation, function, and maintenance of vasculature (Simons et al., 2016). In most studies, VEGF expression in CMT has been closely correlated with metastasis to lymph nodes, clinical stage, tumor grade, and malignancy (Qiu et al., 2008; Vinothini et al., 2009; Clemente et al., 2010, 2013; Millanta et al., 2010; Klopfleisch et al., 2011; Queiroga et al., 2011a; Carvalho et al., 2015a, 2016b; Moschetta et al., 2015; Mucha et al., 2016). However, a few reports have failed to see such an association (Millanta et al., 2006; Santos et al., 2010, 2014). Unfortunately, most of these studies have not differentiated between VEGF isoforms, which would be an interesting additional information. Interestingly, there is evidence for a strong link between immune cells, such as TAMs, CD3+ T-cells, FoxP3+ T-regs, and mast cells, and VEGF expression with increasing malignancy, suggesting that immune cells influence tumor angiogenesis through secretion of VEGF (Restucci et al., 2002; Im et al., 2011; Raposo et al., 2013; Carvalho et al., 2015a, 2016a). Likewise, expression of VEGFR-2, the main signaling VEGF receptor in vascular endothelial cells, in endothelial cells within the tumor tissue increased with malignancy, histological grade, and lymph node metastases, implicating VEGF and VEGFR-2 in angiogenesis in CMTs (Restucci et al., 2004; Diessler et al., 2016; Anjos Dos et al., 2019). One study failed to find a connection between VEGFR-2 expression and histologic grade (Al-Dissi et al., 2010). Interestingly, a positive association between expression of VEGFR-2 and stromal MMP9 has been described, indicating a link between ECM remodeling and endothelial cell activation (Santos et al., 2014).

While expanding our understanding of CAS in CMTs, all these studies have only investigated a very limited number of targets, mostly due to methodological limitations. When information is available as to whether a molecule is expressed in the tumor cells or rather one of the stromal components, it has been mostly obtained through IHC analysis, whereas other approaches have relied on bulk tumor analysis. A major draw-back of the targeted analyses is that one can only analyze targets that are known *a priori*, which precludes unbiased identification of novel molecules of interest. Furthermore, the limited number of targets that can be interrogated through most of these approaches makes it impossible to gain a more wide-angled perspective of changes in molecular networks underlying stromal reprogramming in CMTs. As a direct consequence, unbiased cross-species analyses of molecular homologies and differences in CAS between species have therefore been precluded to date. Due to these limitations, it remains largely unknown to what extent stromal reprogramming in canine and human breast cancer are comparable, and what the molecular similarities and differences are. A better understanding of the biology of CAS in canine breast cancer is imperative to both understand how CAS influences growth and progression of CMTs as well as understand whether canine breast cancer really is comparable to the human disease in all of its aspects.

## Toward a More Comprehensive Picture of Stromal Reprogramming in CMTs

**FIGURE 1:**
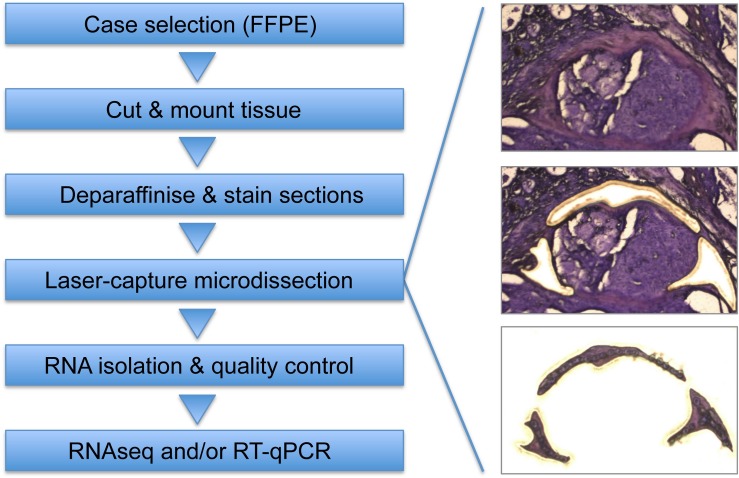
Workflow to isolate and analyze areas of interest from FFPE tissue specimens. After selection of appropriate FFPE specimen, tissue is cut into 10 μm sections and mounted on PEN membrane glass slides (Ettlin et al., 2017). Slides are deparaffinized and stained using Cresyl Fast Violet (Amini et al., 2017), followed by LCM to isolate areas of interest (e.g., CAS). Whenever possible, matched control tissue (e.g., normal stroma) is isolated from the same section to minimize differences in sample processing. RNA isolation is performed using a sonication-based protocol combined with protease digestion (Amini et al., 2017), followed by RNA quality control. Analysis of isolated RNA can be performed by RNAseq [routinely using 4 ng RNA as input per run (Amini et al., 2019a)], by RT-qPCR (Ettlin et al., 2017), and possibly other applications that allow RNA analysis.

Driven by the lack of detailed characterization of stromal reprogramming in CMTs caused by technical limitations described above, we established a workflow to isolate subsections of formalin-fixed paraffin-embedded clinical tumor samples by laser-capture microdissection, and analyze gene-expression changes therein. In a first study, we isolated CAS and matched “normal” stroma (i.e., stroma isolated adjacent to unaltered mammary glands) from FFPE specimen of 13 cases of canine simple mCA, and analyzed the expression of seven well-described CAS-markers in human mCA (PDGFRβ, MMP2, Col1α1, FAP, ACTA2/αSMA, CXCL12/SDF1, and IL6) by RT-qPCR (Ettlin et al., 2017). Our results demonstrated that ACTA2, COL1A1, and FAP were upregulated in canine CAS, while PDGFRB, MMP2, and IL6 expression did not significantly change between normal stroma and CAS. CXCL12 expression was downregulated in CAS compared to normal stroma. IHC validation of these results revealed upregulation of αSMA, FAP, PDGFRB, and Cav-1, while SDF1, MMP2, and FGF2 expression did not change. These findings not only suggested the presence of molecular similarities in CAS biology between canine and human mCA, but also revealed some differences. While interesting, this RT-qPCR-based approach had two major limitations: (i) the targets of interest have to be defined *a priori*, which precludes an unbiased analysis of the samples, and (ii) to the small amount of RNA that can be extracted from small LCM-subsections of FFPE strongly limits the number of RT-qPCR reactions that can be run, thus strongly restricting the number of targets that can be analyzed per sample. To overcome these problems, we further optimized the RNA extraction protocol for the LCM samples of FFPE tissue in a way that increased the average yield per sample between 8- and 12-fold and allowed us to perform next-generation RNAseq (Amini et al., 2017). An overview of the entire workflow is depicted in Figure 1. Thus far, we have successfully applied this novel approach to analyze stromal reprogramming in several different cohorts of clinical samples, including malignant canine mCA and benign canine mammary adenomas. In the following, I will shortly summarize the main findings from these analyses.

### Stromal Reprogramming in Canine Simple Mammary Carcinoma

To begin to understand stromal reprogramming in canine simple mCA on a transcriptome-wide scale, we analyzed matched CAS and normal stroma from 15 clinical cases using our LCM-RNAseq pipeline (Amini et al., 2019a). Strikingly, differential gene expression changes clearly differed between normal stroma and CAS, with 884 significantly deregulated genes. Strongest changes were found in the genes involved in the immune system, cell adhesion and differentiation, ECM organization, and angiogenesis. Clearly, all of these processes are strongly associated with stromal biology, further validating our analytical approach. Unsupervised clustering of samples based on the landscape of immune and stromal cells present in the samples again clearly separated CAS and normal stroma, and revealed strong increases in mesenchymal stem cells, gamma delta T-cells, macrophages, plasmoid dendritic cells, and natural killer T-cells in CAS. These results provide evidence for wide-ranging stromal reprogramming in canine mCA, enabling for the first time a detailed molecular analysis of CAS in canine mCA. We envisage these data to significantly support the understanding of the biology of canine mCA.

### Stromal Reprogramming in Canine Simple Mammary Adenoma

While canine simple mCAs are classified as malignant epithelial neoplasms that infiltrate the surrounding tissue, canine simple mammary adenomas represent benign, well-demarcated, and non-infiltrative tumors that generally contain only very little fibrovascular supporting stroma (Goldschmidt et al., 2011). To date, it remains unclear whether and to what extent stromal reprogramming occurs in these naturally occurring benign tumors of the mammary gland. In fact, stromal reprogramming in human breast cancer has been studied during progression from *in situ* to *invasive* human mCA, in pregnancy-associated breast cancer, in response to therapeutic radiation, and in inflammatory breast cancer (Finak et al., 2006, 2008; Boersma et al., 2007; Casey et al., 2008; Ma et al., 2009; Westbury et al., 2009; Planche et al., 2011; Knudsen et al., 2012; Vargas et al., 2012; Harvell et al., 2013). However, we are not aware of any published dataset regarding stromal reprogramming in naturally occurring benign tumors of the mammary gland. Since CAS has been shown to have important roles in determining the growth and progression of different tumor types, we hypothesized that differences in stromal reprogramming between benign adenomas and malignant mCA could contribute to the clinical behavior of these two tumor types. To begin to understand stromal reprogramming in naturally occurring benign tumors, we thus applied our approach to isolate and analyze CAS and normal stroma from FFPE tissue sections to 13 cases of canine simple mammary adenoma (Amini et al., 2019b). We observed clear separation of normal stroma and CAS samples, and identified 193 genes to be significantly deregulated between the two entities. The strongest changes occurred in processes related to cell adhesion, immune system, proliferation and growth, differentiation, and ECM and collagen organization. Hence, these results demonstrate that substantial stromal reprogramming occurs also in small, benign tumors of the mammary gland.

**FIGURE 2:**
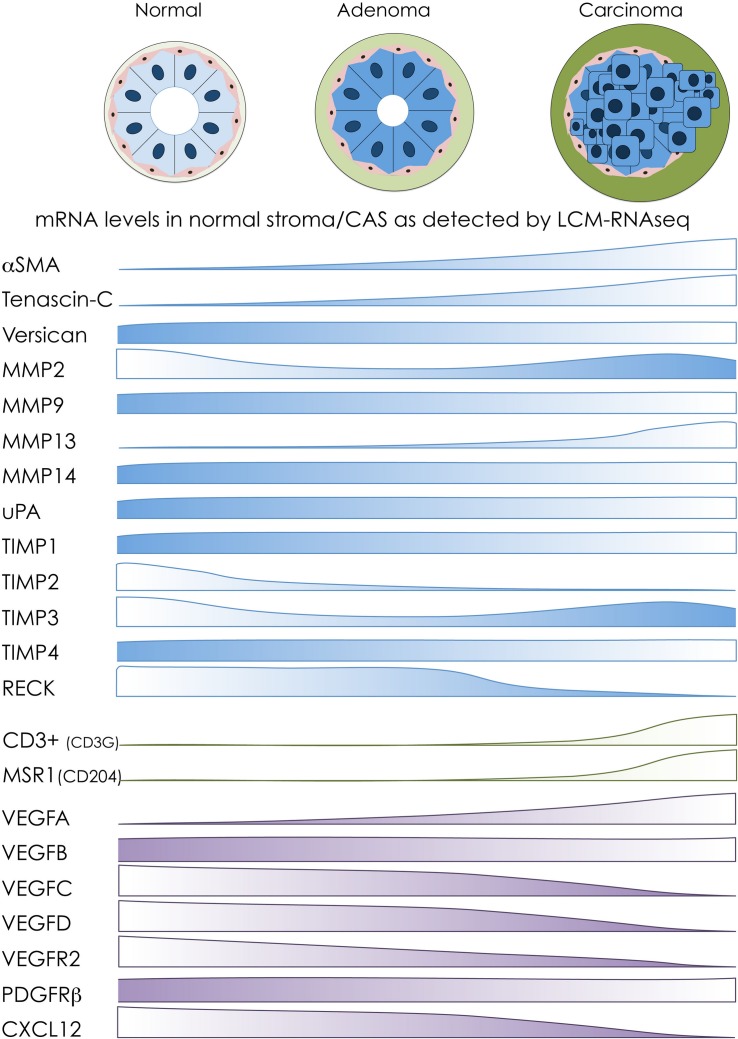
Overview of transcriptomic changes in selected markers during stromal reprogramming in canine mammary adenomas and carcinomas. Schematic overview of transcriptomic changes of selected CAS-related markers based on our transcriptomic analyses of normal stroma, CAS from adenoma, and CAS from carcinoma isolated from canine breast tumor specimens (Amini et al., 2019b). Protein names on the left are targets whose expression in canine tumors has been investigated on protein level mostly by IHC (for details and references, see the main text). The bars next to the protein names indicate changes in RNA abundance of the respective targets between stroma from normal glands **(left)**, stroma from adenomas **(middle)**, and stroma from carcinomas **(right)**, as detected by LCM-RNAseq. Targets associated with fibroblast activation and remodeling of extracellular matrix are depicted in blue, markers of immune cells are colored in green, and targets involved in angiogenesis are shown in purple.

Having previously characterized stromal reprogramming in canine mCA, we then sought to understand commonalities and differences in stromal reprogramming between benign mammary adenomas and malignant mCA. Our analyses showed that CAS in benign adenomas is clearly distinct from malignant mCA. Furthermore, adenoma-derived stroma was much more similar to normal stroma than CAS from mCA, suggesting gradual changes from normal to benign to malignant CAS to occur during the development of tumors. Nevertheless, we also identified commonly regulated genes in CAS of both benign and malignant tumors. This comparative dataset allowed us to interrogate for the first time the transcriptional levels of targets that have been implicated in stromal reprogramming of canine breast tumors thus far (see the sections “Cancer-Associated Fibroblasts and the Extracellular Matrix,” “Infiltrating Immune Cells,” and “Angiogenesis” for details). Figure 2 intends to give a schematic overview of changes in mRNA abundance of these targets between normal stroma, CAS in adenoma, and CAS in carcinoma as detected by our RNAseq approach (Amini et al., 2019b). These data give rise to several interesting observations: (i) some targets, such as αSMA, Tn-C, and VEGFA, show changes in mRNA levels that mirror closely results obtained on protein level, with increasing abundance from normal stroma to adenoma to carcinoma; (ii) a number of targets whose expression has been positively correlated with increasing malignancy (e.g., versican, MMP9, and MMP14) show no changes in stromal mRNA abundance between the three entities; (iii) there are some genes whose expression is opposite of what would be expected from literature regarding protein levels (e.g., MMP2, MMP13, TIMP3, and VEGFR2); and (iv) one of the advantages of RNAseq-based analysis is differentiation between closely related isoforms, e.g., changes in VEGF that are very specific to the different isoforms of the protein. Differences in mRNA versus protein levels can be explained by two different mechanisms: either the main source of the protein in question is not the stroma itself, but rather the tumor cells which release the product into their surroundings. Or, if indeed it is produced by stromal cells, the increase in protein production is due to post-transcriptional regulatory mechanisms that do not impinge on mRNA transcription. To further clarify these aspects, it would be interesting to compare RNAseq data from the tumor cells to that of the respective stroma, or to analyze the different tumor compartments using proteomic analysis pipelines.

### Highly Conserved Stromal Reprogramming Between Canine and Human Mammary Carcinoma

Our next aim was to understand to what extent stromal reprogramming in canine and human mCA is comparable. We reasoned that if stromal reprogramming in the two species shared high levels of homology, this should result in a similar expression pattern of differentially regulated genes between normal stroma and CAS in both. We assessed this using several different approaches. Firstly, juxtaposition of our canine CAS dataset to a similar human dataset revealed that genes upregulated in the canine dataset were on average also upregulated in the human dataset, and likewise genes downregulated in the canine dataset were also downregulated in the human dataset. Secondly, we ranked the samples in the TCGA breast cancer subset (that contains >1000 human tumor samples) according to stromal enrichment scores (i.e., according to how much stroma they contain) to compare our canine-derived stromal signature with. By doing so, we found the canine-derived stromal signature to be highly positively correlated with the enrichment of human-derived stromal signature of the TCGA breast cancer subset. The commonly perturbed pathways between canine and human CAS included angiogenesis, epithelial mesenchymal transition, glycolysis, pathways involved immune response, and others. And finally, we demonstrated that the high level of molecular homology between canine and human stromal reprogramming manifested in a prognostic value of the canine CAS signature, with upregulated genes in canine CAS highly enriched among adversely prognostic genes in humans, and upregulated genes in canine normal stroma highly enriched among favorably prognostic genes in humans. In conclusion, these results clearly demonstrated that stromal reprogramming in canine and human mCA shares significant molecular homology. This homology derives from conservation of key signaling pathways which underlie the prognostic value of stromal gene expression changes in both canine and human mCA. Hence, these findings clearly emphasize the value of canine mCA as a model for human mCA.

## Conclusion and Outlook

Increased understanding of stromal reprogramming in tumors requires the ability to selectively analyze patient-derived CAS, ideally using untargeted methods, such as RNAseq. To date, stromal reprogramming has been mostly investigated using laser-capture microdissection of fresh-frozen tissue, coupled with microarrays or sequencing approaches. However, use of fresh-frozen tissues most often necessitate establishing prospective trials to collect samples accordingly, requiring a high grade of coordination between surgical resection and analysis, and introducing temporal until all required cases are collected. More importantly, it also precludes the analysis of archival FFPE samples, which are the standard product of any pathology department and can be kept at room temperature over long periods of time. To circumvent these problems, we have developed a protocol that allows the analysis of subsections of FFPE patient samples by RNAseq, and have demonstrated its feasibility and usefulness by analysis of stromal reprogramming in several cohorts of patient samples. Importantly, the protocol can be adapted to interrogate transcriptional reprogramming of any area of interest in any type of tissue of any organism, provided that the area is sufficiently large to be isolated and contains sufficient RNA. Hence, we hope our approach to enable a wide range of projects to understand transcriptional reprogramming within distinct compartments of entire tissues. Over the last few years, technological advances have made it possible to analyze tumor (and other) tissues on the cellular level by single-cell RNAseq. This presents a tremendous advance in analytic power and novel insights. However, single-cell RNAseq can only be performed on fresh tissue, which again precludes its applicability for analysis of archival FFPE tissue. Also, the currently available methodology is no accurate enough yet to be routinely used in clinics. Furthermore, it requires sometimes lengthy digestion steps to dissociate tissues into single cells that quite possibly also introduce a fair amount of gene expression changes during the preparation. Finally, the cost of single-cell RNAseq experiments still is substantially higher than that of “canonical” RNAseq analyses. Due to all these aspects, analysis of tissue subsections of FFPE tissues using our LCM-RNAseq approach presents a complementary approach of great value to further understand transcriptional changes in defined locations of clinical specimen.

Despite the advances in analysis of stromal reprogramming that occurs in human tumor samples, the study of CAS in canine tumors has strongly lagged behind. Although a subset of molecular and cellular aspects have been relatively well studied in CMTs, the field is still far from having a more wide-angled overview of stromal reprogramming that occurs in these tumors. For the first time, our studies have started to shed light into stromal reprogramming in canine simple mCA and canine simple mammary adenoma, and begun to analyze the extent to which CAS in canine mCA and human mCA compare. Our data show wide-ranging stromal reprogramming in both canine mCA and adenoma, and also reveal strong molecular homologies between stromal reprogramming in human and canine tumors. Further in-depth analysis of these data have the potential to significantly increase our understanding of stromal reprogramming in canine mCA, and also to identify the conserved aspects between species that are likely drivers of the disease. Better understanding of the molecular underpinnings of canine and human CAS holds enormous potential for further interesting findings.

## Author Contributions

The author designed and wrote the entire manuscript without external help.

## Conflict of Interest

The author declares that the research was conducted in the absence of any commercial or financial relationships that could be construed as a potential conflict of interest.
